# Comparison of Reproductive Strategies between Two Sympatric *Copsychus* Passerines

**DOI:** 10.3390/ani14040554

**Published:** 2024-02-07

**Authors:** Ziqi Zhang, Jianli Bi, Xu Zhao, Yan Cai, Canchao Yang

**Affiliations:** Ministry of Education Key Laboratory for Ecology of Tropical Islands, College of Life Sciences, Hainan Normal University, Haikou 571158, China

**Keywords:** life history, nest-site selection, egg incubation, nestling feeding, niche differentiation

## Abstract

**Simple Summary:**

The fate and development of birds largely depend on their reproductive strategies, which vary, either diverging or converging among different species and populations. We studied the reproductive strategies of White-rumped Shama (*Copsychus malabarica*) and Oriental Magpie Robin (*C. saularis*), which may be convergent because of their kin relationships or divergent because of niche differentiation under sympatric competition. We observed that they differed in certain reproductive strategies, such as nest-site selection and egg incubation patterns, but were similar in others, including nestling heating, sexual synchronization in nestling feeding, and coordination of nestling feeding and nest cleaning.

**Abstract:**

Reproduction plays a crucial role in determining the development, fate, and dynamics of bird populations. However, reproductive strategies vary among species and populations. In this study, we investigated the reproductive strategies of the Oriental Magpie Robin (*Copsychus saularis*) and White-rumped Shama (*C. malabarica*), which are closely related passerines that reproduce in sympatric areas. We found that although these two species were both cavity nesting, their nest-site selection differed; the Shama preferred nesting close to trees and forests, whereas the Magpie Robin nested close to human residential areas. Furthermore, their egg incubation patterns differed; the Shama increased daily incubation frequency with incubation time, but the Magpie Robin maintained its daily incubation time regardless of changes in incubation frequency. However, the nestling heating patterns of these two species were similar, indicating a critical demand for regulating hatchling body temperature during this crucial stage. The feeding frequencies of male parents were strongly correlated with those of females in both species, suggesting equal contribution and good synchronization between the sexes. Nestling feeding frequency was also correlated with nest cleaning frequency, implying coordination between feeding and defecation by parents and offspring, respectively. This research explored the divergence and convergence of reproductive strategies between these two sympatric species, providing valuable insights into the niche differentiation theory.

## 1. Introduction

Life history encompasses the processes of growth, development, and reproduction throughout an organism’s lifetime and constitutes a pivotal step in the evolutionary trajectories of biological entities [1]. Within this context, reproduction is a key driver of biological evolution, as reproductive strategies profoundly shape the contribution of an organism to the genetic heritage of its progeny [2]. Birds expend considerable time and energy on various breeding activities, including mate selection, breeding territory determination, nesting behavior, laying, and brooding behavior [3]. However, numerous factors influence avian reproductive success, such as nest-site selection [4,5], nest predation [6,7], brood parasitism [8], food resources [9], and phenological conditions [6,10]. Wild birds are increasingly coming into contact with human activities, which in turn increasingly exert significant impact on avian reproduction [11,12]. Therefore, investigating factors that affect bird reproduction can establish the theoretical groundwork for understanding the evolution of life-history diversity in birds, thereby contributing to the development of an avian life-history theory [13].

According to niche theory, coexisting species are expected to mitigate interspecific competition by partitioning shared resources, a process that may simultaneously favor the development of phenotypic differences [14]. Ecological niche competition can manifest as direct competition for resources such as forage, habitats, or nesting sites [15,16,17]. It can also manifest as indirect competition, for instance, in the forms of varied temporal utilization of ecological niches or behavioral differences, to mitigate direct competitive pressures [18,19]. Under limited spatial and food resources for breeding, avian species are compelled to engage in competitive interactions as survival strategies [20,21]. Therefore, bird species coexisting in ecological niches and experiencing resource competition in the same habitat have evolved distinct reproductive strategies, including differentiation of nest-site preferences [22,23,24], breeding timing, and nest material utilization [25,26,27,28] to enhance their respective fitness and facilitate coexistence within the same habitat [19]. Among the forms of competition above, competition for nest sites could be particularly intense among secondary cavity-nesting birds, as the availability of suitable secondary nest sites in the same area is limited [29]. Additionally, a favorable nest site not only withstands adverse weather conditions but also protects eggs and nestlings against being preyed upon by predators [30].

To enhance competitive fitness, most avian species have evolved incubation and parental care behaviors [31]. Incubation behavior is an energy-demanding activity for parent birds; it maintains optimal incubation temperatures for embryos and reduces threats from predators, thereby enhancing the reproductive success of offspring [32,33]. Consequently, parental birds must strike a balance between the time allocated to egg incubation and foraging activities [34,35], thereby optimizing their own fitness. Nestling heating occurs in the early stages after the eggs hatch when the hatchlings are fragile with little need for food; therefore, the incubating parent continues sitting in the nest to maintain the hatchling’s body temperature [36]. During the feeding period, the parents feed the nestling for growth and development. The feeding frequency varies according to species, populations, and individuals, and the parents need to make a tradeoff between finding food for their nestling and their own for survival [37]. Nest sanitation refers to the removal of various objects from the nests by the parents, including dropping litter, eggshell debris, fecal sacs, invertebrate parasites, occasionally dead nestlings, and unhatched eggs [38,39]. This behavior reduces the risk of attracting predators, protects eggs from physical damage, and keeps the nests warm and dry, and is thus beneficial for the health of both adults and nestlings [40]. During their long-term evolution, birds have developed a series of behavioral and physiological strategies to ensure parental survival and maximize the benefits of parental investment [41].

Here, we compared the reproductive strategies of two *Copsychus* passerines, the White-rumped Shama (*C. malabarica*) and the Oriental Magpie Robin (*C. saularis*). Previous studies have preliminarily reported the reproductive biology of the Shama in Hawaii, USA [42], the effects of roads on the success rate of Shama nesting and nestling survival, and the effects of vegetation cover on nesting-site selection and nesting success in Thailand [43,44]. Research on Magpie Robins primarily focuses on nesting comparison between nest boxes and burrows, and parent feeding patterns of nestlings [45,46]. Magpie Robins and Shamas are closely related, belonging to the same genus and being sympatric in breeding in the current study area. To achieve long-term coexistence, these two bird species may have undergone divergent evolution in their reproductive strategies. The aim of this study is to provide a comprehensive foundation of reproductive ecology for two species of *Copsychus* passerines by comparing various reproductive strategies, including the breeding cycle of nest building, egg laying, egg incubation, nestling heating, nestling feeding, and nestling growth. The findings will elucidate the evolutionary changes in reproductive characteristics in birds and aid in verifying and exploring theories related to ecological niche differentiation.

## 2. Materials and Methods

### 2.1. Study Area

**Figure 1:**
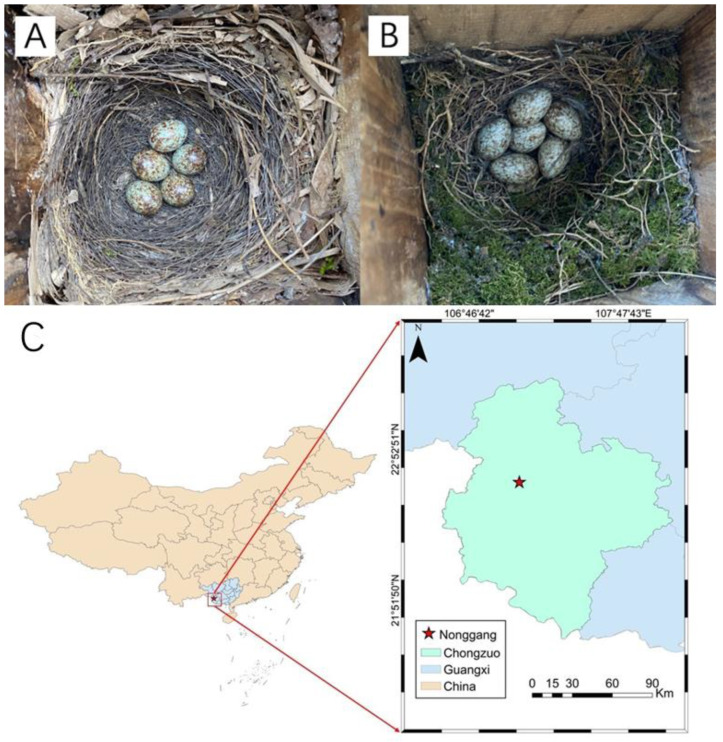
Nests and eggs of White-rumped Shamas (**A**), Oriental Magpie Robins (**B**), and location of the Nonggang village in Guangxi, Southwest China (**C**) [107°04′54″ E, 22°39′09″ N].

The study area is located in the Nonggang village of Guangxi, Southwest China (107°04′54″ E, 22°39′09″ N) (Figure 1). It encompasses a lengthy strip of land stretching from northwest to southeast, spanning a total length of 33.53 km from west to east. The study area comprises the Longhu, Nonggang, and Longshan regions and covers a total area of 10,409.4 ha. The site is situated within the typical Garst karst landform at elevations ranging from 300 to 700 m [47]. In January, the average temperature stands at 13.8 °C, while, in July, it reaches 28.1 °C. The annual average temperature ranges between 20.8 and 22.4 °C, with an annual rainfall of approximately 1150 to 1550 mm [48]. The predominant vegetation types in this region encompass broadleaf forests, cultivated areas, shrublands, bamboo forests, and orchards featuring fruit trees. Notably, extensive sugarcane (*Saccharum officinarum*) cultivation dominates the open landscapes, serving as a primary agricultural crop in the area [49]. In this region, potential nest predators include snake predators, Indochinese Green Magpie (*Cissa hypoleuca*), White-winged Magpie (*Urocissa whiteheadi*), and Greater Coucal (*Centropus sinensis*) [50].

### 2.2. Study Species

Both the Oriental Magpie Robin and White-rumped Shama belong to the genus *Copsychus* in the family Muscicapidae of Passeriformes [51]. They are both cavity nesting with similar nests and eggs (Figure 1). Magpie Robins are distributed in Eurasia, the Indian subcontinent, the Indochina Peninsula, the Wallace Region, and the Pacific Islands [51]. They normally nest in holes in trees, walls, caves, nestboxes, and gaps in the eaves of buildings and branches of trees [46]. The Magpie Robin has a broad range compared with that of the Shama. Research has primarily focused on the song performance rules (clear-cut alternation of acoustic patterns and pauses) [52], geographical sound differences [53], the impact of urbanization on sound [54], and species identification [55]. Shamas are mainly distributed in Southeast Asia, including China, India, Nepal, Bhutan, Bangladesh, Sri Lanka, Myanmar, Vietnam, Laos, Thailand, Malaysia, and Indonesia [56]. They nest in bamboo or tree holes, with records of nesting in artificial nest boxes as well, and use plant leaves, catkins, stems, roots, and other parts as nesting materials [42,56]. Research on the White-rumped Shama includes observations on the nest, egg size, and predation behavior of the Yunnan population [56]. Other studies have investigated its involvement in illegal trade and conservation efforts [57], nest-site selection [58], nest defense behavior [59], and population size and distribution [60].

### 2.3. Field Procedure

The present study was performed during the breeding season (April–August) of 2019 and 2020. The artificial nest boxes were made from Chinaberry (*Melia azedarach*) wood and were 30 cm in height and 15 cm^2^ at the bottom. The entrance hole in the front of each nest box was 6 cm in diameter. In 2019, 191 nest boxes were deployed, and positioned 3 m above the ground, either on trees or utility poles. The selection of nest box sites extended from the local residential areas of the village to the forest. Each nest box was located using GPS to measure its distance to the road and forest, while the orientation of the entrance was determined using a compass. The nest boxes were monitored every 2–3 days to investigate their occupation status. When nest materials were identified in the nest boxes, some were monitored daily using mini cameras (HD99S-32G; Sig Technology Co., Ltd., Shenzhen, China) to avoid physical interference, while the others were investigated regularly, recording the following parameters: (1) nest building stage (day in unit), (2) clutch size (egg number of complete clutch), (3) egg incubation stage (day in unit), and (4) nestling feeding stage (day in unit). According to our observations, neither camera installation nor researcher nest visiting caused unusual nest desertions. Egg and body mass were weighed using an electronic scale (CX-668B; Changxie Electronics, Wuxi, China; accurate to 0.01 g). Egg size was calculated as egg volume according to the formula: egg volume = 0.51 × egg length × (egg width)^2^ [61], while nestling growth was monitored after hatching, and the recorded parameters included body mass and tarsus length. Egg length, width, and tarsus length were measured using a dial Vernier caliper (WiHa Inc., Eisenach, Germany; accurate to 0.02 mm).

Using video playback, we meticulously documented incubation duration, nestling heating duration, feeding duration, nest cleaning duration, and departure from the nest, and quantified several reproductive behaviors. Egg incubation frequency refers to the times eggs are incubated daily by parents during the egg incubation stage; nestling heating frequency refers to the times nestling are warmed daily by parents during the early nestling stage after the eggs are hatched out; nestling feeding frequency refers to the times nestlings are fed daily by parents during the nestling stage; nest cleaning frequency refers to the times feces are cleaned daily by parents during the nestling feeding stage. The primary data (including egg incubation, nestling heating and feeding, and nest cleaning frequencies) of this study were collected using mini cameras to minimize disturbance. Researchers only visited when necessary to confirm specific parameters such as egg-laying initiation and nest fate. Except for the nestling measurements, researchers quickly observed the nest-box contents without unnecessary touching. Furthermore, visits on cavity nests (e.g., nest boxes) were supposed to have little impact on the vegetation around the nests compared with natural nest sites (e.g., bush nests). Therefore, the nest disturbance in this study was controlled appropriately. Nest predation was confirmed when eggs or nestlings were destroyed or predated. This phenomenon was easily identifiable because predated nests were usually accompanied by residues of eggs or nestlings and destroyed traces of the nests. Furthermore, nest desertion was not found in this study.

### 2.4. Statistical Analyses

Data analyses were performed using SPSS for Windows (SPSS Inc., Chicago, IL, USA) or R (v. 4.2.2) for Windows (R Foundation for Statistical Computing, Vienna, Austria). Statistical methods for variable comparisons were selected according to the attributes, distribution, and variance of the data. Tree or wire pole selection and hole orientation of nest boxes between Magpie Robins and Shamas were compared using the chi-square test and Rayleigh test of uniformity, respectively. The distance to the road or forest of the nestboxes between the two studied species was compared using the Mann–Whitney U test because of non-normal distribution of the data (distance to road: Z = 0.279, *p* < 0.001, distance to forest: Z = 0.284, *p* < 0.001, Kolmogorov–Smirnov test). Student’s or Welch’s *t*-test was used to compare other variables, including egg mass and size, clutch size, and time at different breeding stages. Pearson’s or Spearman’s correlation coefficient was used as a parameter or non-parameter test, respectively, for correlation testing according to the data distribution. The data of nestling growth were continuous variables used in the generalized linear model (GLM) to compare the growth of the nestlings for Magpie Robins and Shamas. Nest predation rates for species were compared using a chi-squared test. The method outlined by Nekola and White [53] was used for calculating the statistical inference of the difference in slope (DS) between two regression lines. The GLM and comparison of slopes were performed using the robust [62] and simba [63] packages in R while other analyses were conducted in SPSS. All tests were two tailed, and data are presented as the median ± interquartile range (IRQ) or mean ± standard error (SE). Statistical significance was set at *p* < 0.05.

## 3. Results

### 3.1. Nest Box Selection

Shamas occupied a greater proportion of nest boxes on trees (81.5%), whereas Magpie Robins occupied more nest boxes on poles (80%; Table 1). Furthermore, Shamas used nest boxes much closer to the forest than those used by Magpie Robins (Table 1). However, the distance to the road between Shamas and Magpie Robins did not differ significantly (Table 1). The orientations of nest-box entrances differed significantly between the two species (Table 1). The Magpie Robins exhibited more southerly orientations in the nest-box entrances when compared with those of the Shamas.

### 3.2. Breeding Stages and Clutch Size

**Figure 2:**
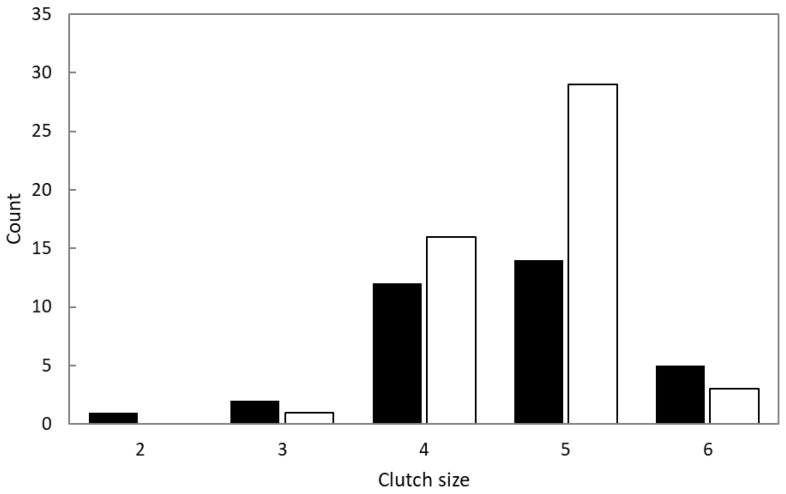
Comparison of clutch sizes between White-rumped Shamas and Oriental Magpie Robins. Black and white bars represent Shamas and Magpie Robins, respectively.

The nest-building stage of White-rumped Shamas was significantly shorter than that of Oriental Magpie Robins (Table 2). For the egg incubation stages, no significant difference was observed between the species (Table 2). However, the nestling feeding stage of Shamas was significantly shorter than that of Magpie Robins (Table 2). The most common clutch size for both Shamas and Magpie Robins was five, followed by four (Figure 2). Clutch sizes of three and six were few for both species, but that of two was only found in the Shamas. The mean clutch size did not differ significantly between the two species (Shama: 4.6 ± 0.2, *n* = 34; Magpie Robin: 4.7 ± 0.1, *n* = 49; Welch’s *t*-test: *t* = −0.58, df = 53.18, *p* = −0.563).

### 3.3. Egg Incubation and Nestling Heating

**Figure 3:**
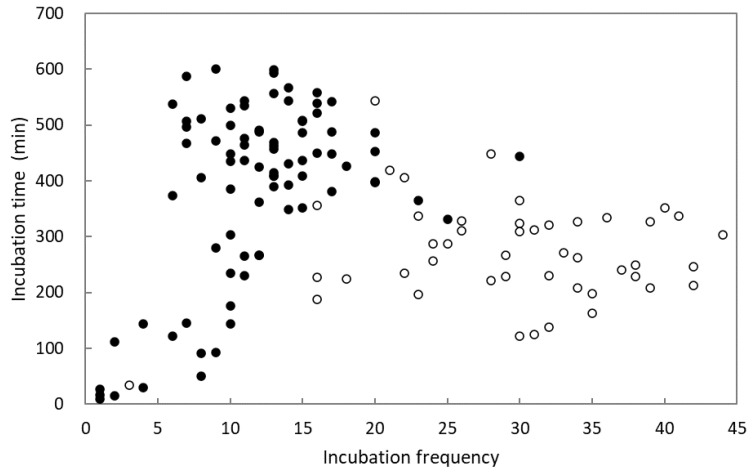
Association between incubation time and frequency per day in White-rumped Shamas and Oriental Magpie Robins. Black and white dots represent Shamas and Magpie Robins, respectively. The sample sizes of Shamas and Magpie Robins were 82 days from seven nests and 46 days from four nests, respectively.

**Figure 4:**
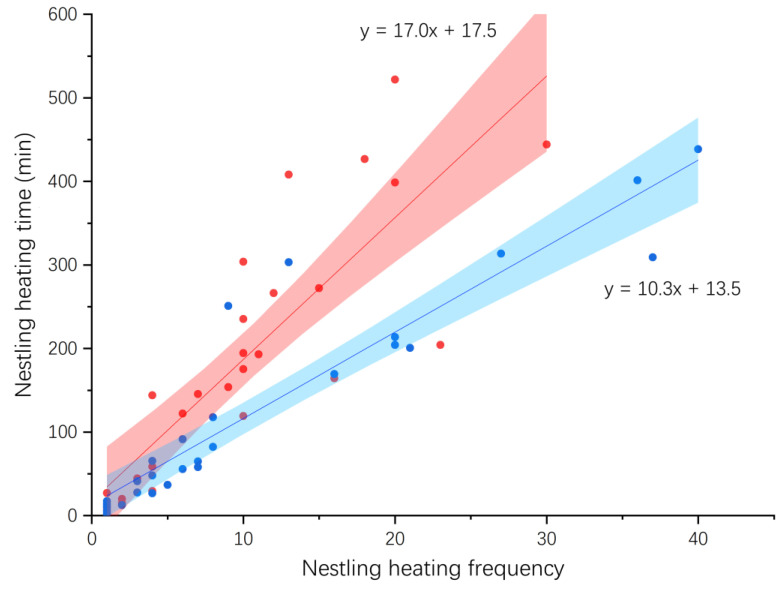
Association between nestling heating time and frequency per day in White-rumped Shamas and Oriental Magpie Robins. Red and blue dots/lines represent Shamas and Magpie Robins, respectively. Shaded areas refer to 95% confidence interval. The sample sizes of Shamas and Magpie Robins six and four nests, respectively, for 28 days.

The mean egg-laying periods of Shamas and Magpie Robins lasted 99 and 107 days, respectively, with no significant difference between them (1 = March 25; Shama: 56.70 ± 4.65; Magpie Robin: 48.19 ± 3.47; Student’s *t*-test: *t* = 1.49, df = 93, *p* = 0.140). According to 82 days of video data from seven nests of Shamas at the egg incubation stage and 46 days from four nests of Magpie Robins, the incubation frequency per day was significantly correlated with the incubation time per day in Shamas (Pearson correlation: *r* = 0.49, *p* < 0.001, *n* = 82), but not in Magpie Robins (Pearson correlation: *r* = 0.04, *p* = 0.796, *n* = 46). The relationship between incubation frequency and time for Shama was linear (Figure 3). Egg incubation was only performed by females in both Shamas and Magpie Robins, with males bringing food to females during incubation in both species. Furthermore, according to 28 days of video data on nestling heating from six Shamas and four Magpie Robin nests, the nestling heating frequency and time were significantly correlated in both species (Shama: Pearson correlation: *r* = 0.85, *p* < 0.001, *n* = 28; Magpie Robin: Spearman correlation: *r* = 0.96, *p* < 0.001, *n* = 28; Figure 4). However, the slopes of regression lines differed significantly between the two species (DS = 6.66, *p* = 0.007).

### 3.4. Nestling Feeding and Growth

**Figure 5:**
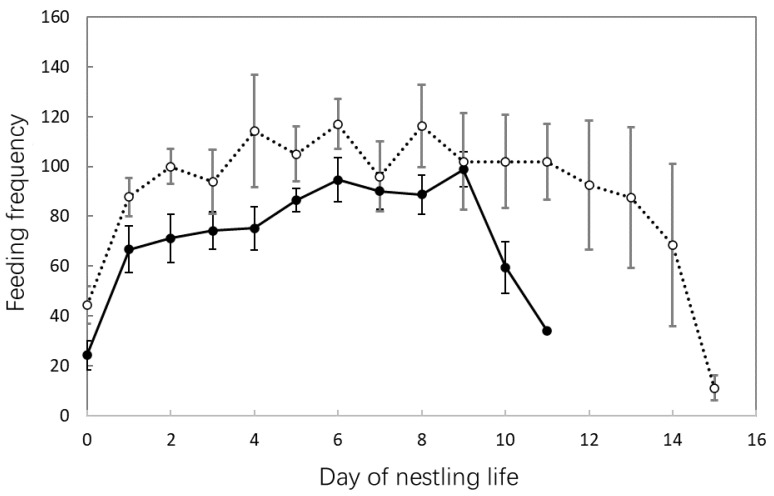
Feeding frequencies of nestling per day in White-rumped Shamas and Oriental Magpie Robins. Black dots/solid line and white dots/dashed line represent Shamas and Magpie Robins, respectively. Error bars represent SE. The sample sizes of Shamas and Magpie Robins were 67 days from six nests and 61 days from four nests, respectively.

**Figure 6:**
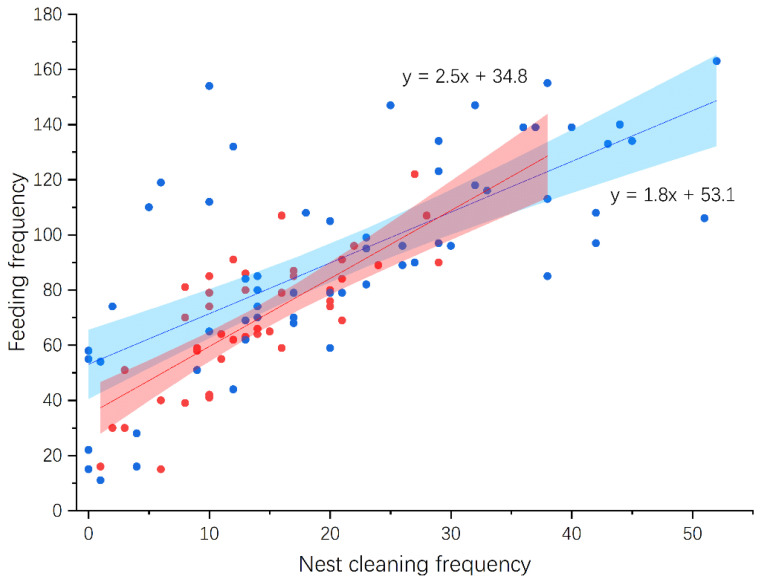
Association between feeding and nest cleaning frequencies per day in White-rumped Shamas and Oriental Magpie Robins. Red (y = 2.5x + 34.8) and blue (y = 1.8x + 53.1) dots/lines represent Shamas and Magpie Robins, respectively. Shaded areas refer to 95% confidence interval. The sample sizes of Shamas and Magpie Robins were 43 and 61 days from four nests, respectively.

**Figure 7:**
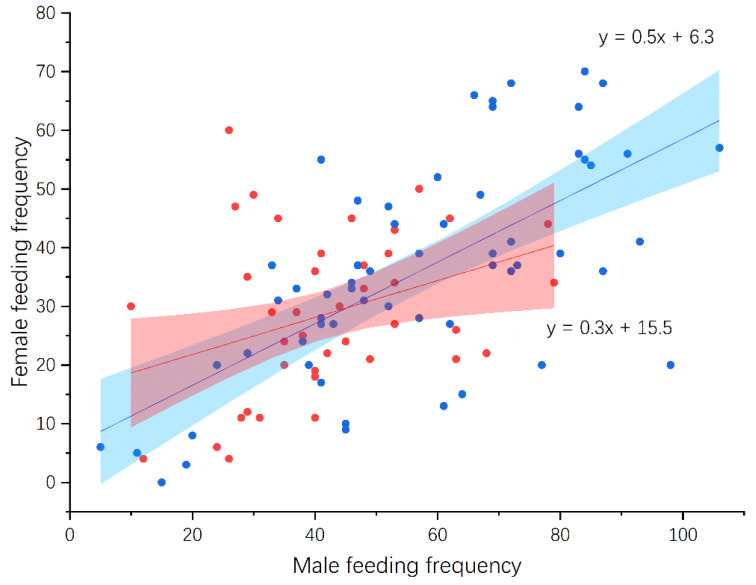
Association between male and female feeding frequencies in White-rumped Shamas and Oriental Magpie Robins. Red (y = 0.3x + 15.5) and blue (y = 0.5x + 6.3) dots/lines represent Shamas and Magpie Robins, respectively. Shaded areas refer to 95% confidence interval. The sample sizes of Shamas and Magpie Robins were 43 and 61 days from four nests, respectively.

According to video data spanning 67 and 61 days from six White-rumped Shama and four Oriental Magpie Robin nests, respectively, the feeding frequencies of Shamas were lower than those of Magpie Robins throughout the nestling feeding stage (Figure 5). The feeding frequencies of Shama and Magpie Robins began to decrease from the 9th and 11th day, respectively. Feeding frequency was positively correlated with nest cleaning frequency in both species (Pearson correlation: Shama: *r* = 0.79, *p* < 0.001, *n* = 43; Magpie Robin: *r* = 0.70, *p* < 0.001, *n* = 60; Figure 6), and no significant difference was observed between their slopes of regression lines (DS = 0.63, *p* = 0.081). Moreover, the feeding frequencies between males and females in Shamas (Pearson correlation: *r* = 0.35, *p* = 0.020, *n* = 43) and Magpie Robins (Pearson correlation: *r* = 0.66, *p* < 0.001, *n* = 60; Figure 7) were correlated. Similarly, no significant difference was detected for slopes of regression lines between species (DS = −0.21, *p* = 0.094). Regarding nestling growth, both body mass and tarsus length changed according to nestling age (GLM: body mass: Z = 76.44, *p* < 0.001; tarsus length: Z = 65.95, *p* < 0.001; Table 3) but were similar between the Shama and Magpie Robin nestlings (GLM: body mass: Z = −0.46, *p* = 0.649; tarsus length: Z = 0.42, *p* = 0.674; Table 3). Finally, the nest predation rates of Shamas and Magpie Robins were 16.0% (*n* = 31) and 3.2% (*n* = 25), respectively, with no significant differences (Chi-square test: χ^2^ = 2.78, df = 1, *p* = 0.096).

## 4. Discussion

The experimental results indicate that the two species of *Copsychus* passerines exhibit distinct preferences in nest-site selection. They also adopt different incubation patterns during the egg stage and employ varied strategies in nestling heating and feeding behaviors during the nestling stage. Consequently, these two species of *Copsychus* passerines have evolved distinct reproductive strategies to facilitate long-term coexistence in sympatric breeding. The more ecological niches overlap, the more similar resources will be used, and limited resources will lead to fierce competition [64]. For secondary cavity-nesting birds, burrow resources are limited, leading to competition, which may further lead to niche differentiation between sympatric species [23]. Our research showed that Shamas preferred to nest in nest boxes on trees and close to forests, whereas Magpie Robins preferred to occupy nest boxes on poles and close to human residences. This was expected because the Magpie Robin is a widespread and adaptable species that dominates both rural and urban areas [65]. The selection of living or breeding sites close to human settlements by Oriental Magpie Robins may be an adaptation to reduce predation risk, as numerous predators are distant from humans [66,67]. However, although the nest predation rate of Magpie Robins seemed to be lower than that of Shamas (3.23% vs. 16%), the difference was not statistically significant. Shamas exhibit a preference for nesting at the forest edges, possibly owing to the relatively higher abundance of insects in that habitat [68,69]. In a study on Blue Tits (*Cyanistes caeruleus*), supplemental food provisioning could reduce their nest-building times [70]. This may explain the shorter nesting period of Shamas relative to that of Magpie Robins in the present study. Furthermore, other studies have indicated that factors such as egg-laying date advancement and nest height influence nest-building time [71], while the type of nest (open nests vs. inbox nests) affects the nest-building time of secondary cavity-nesting birds [72].

Variation in clutch size is multifactorial. Clutch size increases with increasing food abundance [73] and is negatively correlated with predation pressure [74]. In addition, clutch size increases with an increase in latitude [75] and is negatively correlated with altitude [76]. In the present study, the clutch size of the Shamas was similar to that of Magpie Robins; however, smaller eggs were laid. Clutch size did not differ because both species inhabit the same northern tropical region, with no difference in altitude or nest predation rate.

Incubation is one of the critical behaviors via which parent birds improve reproductive success; parent birds trade off egg temperature, embryo development time, and energy expenditure [77]. A previous study identified a diminished correlation between egg size/surface-area-to-volume ratio and incubation period, suggesting limited impact of the factors on the duration of incubation [78]. The incubation time per day changed with change in daily incubation frequency in the Shamas. Such changes were linear indicating that the incubation time per day increased with an increase in daily incubation frequency to a certain extent. Conversely, the trend was not observed in the Magpie Robins, suggesting that daily incubation time is more stable for Magpie Robins, such that they maintain the total daily incubation time, even when incubation frequency is thought to increase or decrease. In other words, the Magpie Robins either reduced the duration of each incubation event when the incubation frequency increased or vice versa. This result offers insight into the different strategies used by Shamas and Magpie Robins to regulate incubation time and frequency. The Shamas employed a relatively longer daily incubation time but lower daily incubation frequency than those of the Magpie Robins, indicating a strategy of longer incubation time with a shorter frequency of nest leaving, similar to that of the Greater Coucal in the same area [79]. In contrast, the Magpie Robins adopted a strategy of short incubation time and elevated frequency of nest departure, similar to Great Tits (*Parus major*) [80] and Green-backed Tits (*Parus monticolus*) [81]. Conway and Martin [82] postulated that minimizing the frequency of nest departure and prolonged incubation could reduce the energy expenditure associated with parent birds warming the eggs. Moreover, reducing nest visits reduces the risk of detection by natural enemies [83].

For immature nestlings whose ability to maintain body temperature is low, brooding is critical for maintaining a constant body temperature [3]. When the nestling hatched, both Shamas and Magpie Robins showed a positive correlation between nestling heating time and frequency per day, suggesting that Magpie Robins change the pattern of regulation between time and frequency compared with that for egg incubation. Similar results were obtained for Long-tailed Tits (*Aegithalos caudatus*), implying that brooding adults may adjust their brooding duration according to their partner’s feeding investment and nestling demands [84]. Nestlings need to be fed by their parents after breaking their shells, and the frequency of food supply increases as they grow [85]. The frequency of feeding as a function of nestling age was similar between Shamas and Magpie Robins, with a reduction in the last few days before fledging. This pattern is consistent with numerous previous studies on Green-backed Tits [81], Long-tailed Tits [84], Yellow-bellied Warblers (*Abroscopus superciliaris*) [86], Blue-tailed Bee-eaters (*Merops philippinus*) [87], and White-crested Laughingthrush (*Garrulax leucolophus*) [88]. In the early stages of brooding, chicks are small and have a low demand for food. Furthermore, a certain amount of yolk supply is stored in the nestlings when they hatch; therefore, a small amount of food can maintain their survival [89]. However, the chicks are less able to regulate their temperature at this point, and thus, the parents warm their chicks and feed them relatively less frequently. As they grow, the parents supply more food to the chicks because their food requirements increase dramatically. Nevertheless, the feeding frequency of parent birds decreased in the late period before fledging, perhaps because (1) the chicks’ signs and external organs were in a low growth or equilibrium state [90], which might prepare the chicks for flight, and (2) the begging intensity of nestlings became weaker with increasing age [91], affecting the feeding frequency of parent birds.

Nest sanitation behavior during nestling feeding plays a crucial role in keeping nests dry and free of parasites, thus reducing predator attraction [38]. The current study revealed a positive correlation between nestling feeding frequency and nest cleaning. This finding is consistent with previous research that consistently demonstrated a strong association between parental feeding behavior and offspring fecal excretion. Quan et al. (2015) found that the nestlings of Red-whiskered Bulbul (*Pycnonotus jocosus*), Sooty-headed Bulbul (*Pycnonotus aurigaster*), and Chinese Hwamei (*Garrulax canorus*) defecated after their parents fed them [92]. Similarly, a study on Green-backed Tits indicated that nest-cleaning frequency was highly correlated with feeding frequency [81]. Such association may also imply that the parents perform these two activities during a single nest visit as an adaptation to minimize the risk of predator detection.

In addition, the feeding frequencies of the male and female parents of both species were positively correlated, indicating a positive feedback loop between males and females during feeding. The feeding frequencies of females and males were highly correlated, indicating that the food supply for both sexes was synchronized. This result is consistent with those of previous studies showing that monogamous females and males participate equally in nestling feeding [93]. Similar feeding rates in both sexes have also been demonstrated in Wire-tailed Swallows (*Hirundo smithii*) [94], Western House Martin (*Delichon urbicum*) [95], and Purple Martins (*Progne subis*) [96]. However, a consistent bias in feeding investment toward females has also been demonstrated in the Prairie Warbler (*Setophaga discolor*) [97] and the Eastern Bluebird (*Sialia sialis*) [98]. Furthermore, studies on the Superb Fairywren (*Malurus cyaneus*) have revealed that there may be a complementary relationship between the sexes, with females feeding more and males feeding less [99]. Similarly, in varied tits, male parent feeding frequency was found to be considerably lower than that of females [91]. Finally, nestling growth data showed that Shama and Magpie Robin chicks had similar masses and tarsus lengths at hatching, but Magpie Robin chicks gained additional weight and tarsus length relative to Shama chicks as they grew. A possible reason for this is that Magpie Robins invested more in nestling feeding with a longer brooding period than that of Shamas because they have a larger body mass.

In this experiment, comparative analysis of nest material types and nest weight characteristics is lacking. Additionally, studying whether parental birds nest singly or cooperatively presents relative challenges. Therefore, whether these factors influence nest-building time is unclear. Future research could focus on exploring these aspects in relatively great detail.

## 5. Conclusions

This study revealed that Shamas and Magpie Robins, which are close in kin relations and sympatric in breeding areas, exhibit niche differentiation in nest-site utilization, with Shamas nesting close to trees and forests, whereas Magpie Robins nest close to human residential areas. A disparity was identified between the nest-building time for these two species, which may be related to differences in nest material acquisition and energy expenditure during transport. Although clutch sizes did not differ significantly, distinct patterns of egg incubation were detected between Shamas and Magpie Robins, reflecting the different strategies they adopted. However, nestling heating presented a similar pattern in both species, indicating a critical need to regulate the body temperature of hatchlings at this crucial stage. Furthermore, the feeding frequencies of male and female parents were strongly correlated in both species, suggesting equal contributions and good synchronization in nestling feeding between the sexes. In addition, the frequency of nest cleaning and nestling feeding in both species were significantly correlated, suggesting coordination between the feeding and defecation of parents and offspring, respectively. Moreover, toward the end of fledging, the frequency of parental feeding gradually decreased, probably because of the request to fledge and/or the decrease in nestling begging intensity. Finally, the relatively short nestling feeding period observed in Shamas may be explained by their smaller body mass than that of Magpie Robins. In summary, this study explored the divergence and convergence in reproductive strategies between Magpie Robins and Shamas, providing valuable insights for better understanding the niche differentiation theory.

## Figures and Tables

**Table 1:** Comparison of nest-box selection between White-rumped Shamas and Oriental Magpie Robins.

	Shama (*n* = 27)	Magpie Robin (*n* = 45)	Statistic *	*p*
Location (count)	22 trees, 5 poles	9 trees, 36 poles	χ^2^ = 26.02	<0.001
Distance to forest (m)	0 ± 0 (Median ± IQR)	5 ± 16 (Median ± IQR)	Z = −5.84	<0.001
Distance to road (m)	4 ± 8 (Median ± IQR)	3 ± 7 (Median ± IQR)	Z = −0.49	0.624
Orientation (°)	222.93 ± 19.87 (Mean ± SE)	183.62 ± 15.29 (Mean ± SE)	Z = 3.22	0.040

* The statistical analyses for tree or pole and orientation were performed using the chi-square test and Rayleigh test of uniformity, respectively; the other analyses were performed using the Mann–Whitney U test. Values are mean ± SE excluding location.

**Table 2:** Comparison of three nest breeding stages between White-rumped Shamas and Oriental Magpie Robins with sample size in brackets.

Breeding Stage	Shama	Magpie Robin	Statistic *	*p*
Nest building	3.1 ± 0.2 (19)	4.3 ± 0.4 (20)	*t* = −2.34	0.025
Egg incubation	11.8 ± 0.2 (20)	12.3 ± 0.2 (21)	*t* = −1.84	0.074
Nestling feeding	11.2 ± 0.2 (17)	13.3 ± 0.3 (19)	*t* = −5.79	<0.001

* The statistical analysis for nest building was Student’s *t* test; the other analyses were Welch’s *t* test. Numeric denotes the mean number of days, with the figure in parentheses indicating the sample size.

**Table 3:** Results of generalized linear model results for comparison of nestling growth between species and nestling age.

Response Variable	Effect	Estimate	SE	Z	*p*
Body mass	(Intercept)	1.72	0.02	75.32	<0.001
	Species	−0.01	0.01	−0.46	0.649
	Nestling age	0.16	<0.01	76.44	<0.001
Tarsus length	(Intercept)	2.38	0.02	126.78	<0.001
	Species	0.01	0.01	0.42	0.674
	Nestling age	0.11	<0.01	65.95	<0.001

## Data Availability

The data presented in this study are available on request from the corresponding author. The data are not publicly available due to authors’ consensus.
